# Implementation of a Sensory Room in a Psychiatric Intensive Care Unit: A Mixed‐Methods Study

**DOI:** 10.1111/inm.70103

**Published:** 2025-07-31

**Authors:** Suzanne Dawson, Amy Hawke, Lemma Bulto, Patricia Whitelaw, Ngoni Jeranyama, Justin Newton Scanlan

**Affiliations:** ^1^ Caring Future Institute Flinders University Bedford Park South Australia Australia; ^2^ Southern Adelaide Local Health Network, Repat Health Precinct Daw Park South Australia Australia; ^3^ Health Evidence Synthesis, Recommendation and Impact (HESRI), School of Public Health The University of Adelaide Adelaide South Australia Australia; ^4^ Faculty of Medicine and Health The University of Sydney Camperdown New South Wales Australia

**Keywords:** comfort room, implementation, psychiatric intensive care unit, sensory approaches, sensory room

## Abstract

Sensory rooms provide a safe space for consumers in inpatient psychiatric settings to self‐manage distress and agitation and potentially reduce the use of restrictive practices. However, sensory rooms are often not available, and there is limited knowledge about strategies that support the implementation. This study explored consumer and clinician experiences of a sensory room in an acute psychiatric intensive care unit (PICU) and examined its impacts on restrictive practice use (seclusion and Pro re nata [PRN] medication). A mixed‐methods approach was employed in the analysis of interviews with consumers and clinicians and routinely collected data pertaining to restrictive practice use. Themes were generated from the qualitative data through deductive analysis against the Theoretical Domain Framework (TDF) and COM‐B to understand the barriers and facilitators to use. Rates of PRN use and seclusion were calculated for three 2‐month periods: prior to the implementation of the room and two time points following implementation. Prominent barriers related to limited opportunity to use the room due to the environmental context (room location) and resources (staffing). Facilitators were clustered around consumer and staff motivation to use the room. There were no significant changes in PRN use over time, and while there was a numeric decrease in the use of seclusion, this did not reach statistical significance. In summary, sensory rooms were valued by consumers and most staff and were considered to provide a safe therapeutic space within the often chaotic and complex treatment environment of a PICU. Strategies for successful implementation are discussed.

## 1 Introduction

Internationally, significant efforts are being made to prevent and reduce the use of seclusion and restraint and promote the use of trauma‐informed care approaches in mental health care settings (WHO 2019). In South Australia, the Chief Psychiatrist published a Standard to ‘Reduce and Eliminate where possible the Use of Restraint and Seclusion’ and promote trauma‐informed practice (Chief Psychiatrist 2021). The Standard states that people accessing emergency departments and/or short stay mental health units should have access to sensory modulation as trauma‐informed treatment modalities. In practice, implementing psychosocial approaches into acute mental health inpatient settings remains challenging. This study reports on an interdisciplinary quality improvement initiative to implement a sensory room in a psychiatric intensive care unit (PICU).

### 1.1 Background

Psychiatric intensive care units are intended to be low‐stimulus, secure and safe spaces for people experiencing behavioural disturbance and increased risk of harm to self or others due to their mental illness (Salzmann‐Erikson 2023). Consumers admitted to a PICU are often young, male, with a schizophrenia diagnosis and legally detained. The complexity of consumers experiencing an acute phase of illness often results in staff using medication, seclusion and restraint to manage behavioural disturbance and agitation (Gerace and Muir‐Cochrane 2019). The negative impact of these coercive practices is well acknowledged and includes physical and psychological harm, increased aggression, poor treatment outcomes (Lohmann et al. 2024) and contradicts nursing values (Eren 2014).

Various strategies and programmes have been implemented internationally to facilitate reducing restrictive practices and promote care that is trauma informed. These include policy and regulatory changes, training and education, implementation of trauma‐informed models of care and patient and family involvement (Oostermeijer et al. 2021; Bowers 2014; Huckshorn 2004). Within this context, there is evidence for, and increased uptake of sensory approaches to assist consumers in employing safe ways to manage distress and agitation (Scanlan and Novak 2015; Fletcher et al. 2019). Sensory approaches align with current mental health values, models and policies (Fletcher et al. 2019) and can be used as an alternative to more restrictive practices (i.e., Pro re nata [PRN] medication/seclusion). Sensory rooms are a purposefully designed therapeutic space used to support sensory‐based therapeutic interventions (Barbic et al. 2019) and have been introduced in psychiatric inpatient settings to improve care and reduce restrictive practices (Oostermeijer et al. 2021), although little is known about their use in PICUs. Hedlund Lindberg et al. (2019) included participants from a PICU setting in their study exploring patient experiences of a sensory room; however, it was not possible to distinguish findings for this participant group.

Most studies on sensory rooms have explored outcomes that measure consumers self‐rated distress (at a particular point in time) and seclusion and/or restraint rates (Scanlan and Novak 2015; Dorn et al. 2020; Novak et al. 2012). In general, sensory rooms have been found to reduce self‐reported distress, although the reduction in seclusion and restraint rates are mixed (Oostermeijer et al. 2021). A recent systematic review that examined the effectiveness of sensory rooms in psychiatric settings reported that one out of four studies reported a reduction in seclusion and one out of two studies reported reduced restraint incidents (Haig and Hallett 2023). Studies that have explored consumer and staff experiences of use identify positive impacts on therapeutic relationships (Barbic et al. 2019; Björkdahl et al. 2016). Whilst clinicians' attitudes to trauma‐informed approaches are favourable, there is limited knowledge and application of such approaches in practice (Cilia Vincenti et al. 2022). Lack of resources and alternative options available have been reported by nurses as barriers to the reduction and elimination of seclusion and restraint (Gerace and Muir‐Cochrane 2019). However, implementing psychosocial and trauma‐informed interventions/approaches into settings with a biomedical focus is challenging, with clinicians highlighting the dominant medical model and emphasis on risk assessment as prominent barriers (Cooke et al. 2019).

Given the challenges in implementing novel psychosocial interventions in inpatient mental health care settings, we were interested in examining uptake of a sensory room in a PICU, where consumers are experiencing an acute phase of their illness and more likely to experience restrictive practices. This study aimed to explore consumers' and clinicians' experiences of a sensory room and determine facilitators and barriers to support uptake in a PICU. The study also examined if use of a sensory room impacts on restrictive practice incidents (seclusion/restraint and PRN medication use).

## 2 Methods

### 2.1 Study Design

A mixed‐methods approach was employed to evaluate the implementation and impact of a sensory room. Two frameworks were used to understand the barriers and facilitators to implementation: the Theoretical Domains Framework (TDF) which describes the factors that affect behavioural change (e.g., knowledge, skills and optimism) and the Capability, Opportunity, and Motivation–Behaviour (COM‐B) model to understand the mechanisms that lead to behaviour change (Michie et al. 2011). These frameworks are often used in combination to identify and describe the prominent enablers and barriers and the most effective intervention strategies to support change in practice/behaviour (e.g., social influences, education) (Cane et al. 2012). The study followed the Revised Standards for Quality Improvement Reporting Excellence (SQUIRE 2.0).

### 2.2 Setting

The setting was an 8‐bed PICU, located within a larger psychiatric inpatient facility in Adelaide. The PICU offers short‐term psychiatric care for adults experiencing an acute phase of mental illness, including those with psychosis, mood and anxiety disorders and personality disorders. The multidisciplinary team, including nursing, psychiatry, occupational therapy, social work and lived experience staff, provide therapeutic assessment, collaborative care planning and interventions, working towards transfer of care to a less restrictive environment (often an adjoining inpatient ward). Nurses are the only staff group who work on the unit over 24 h. The nursing:patient ratio is 5:8 during the day and 4:8 during the night.

### 2.3 Context for the Study

In late 2021, a sensory room attached to the PICU was opened. Due to space constraints on the ward, the room was established from an office space situated off an adjacent corridor. There is a new contemporary purpose‐built 12‐bed PICU in the building phase, which will include a sensory room but replace the current PICU, which is unsuitable in design. The sensory room was an attempt to improve the therapeutic space and options for consumers.

### 2.4 Intervention

**FIGURE 1:**
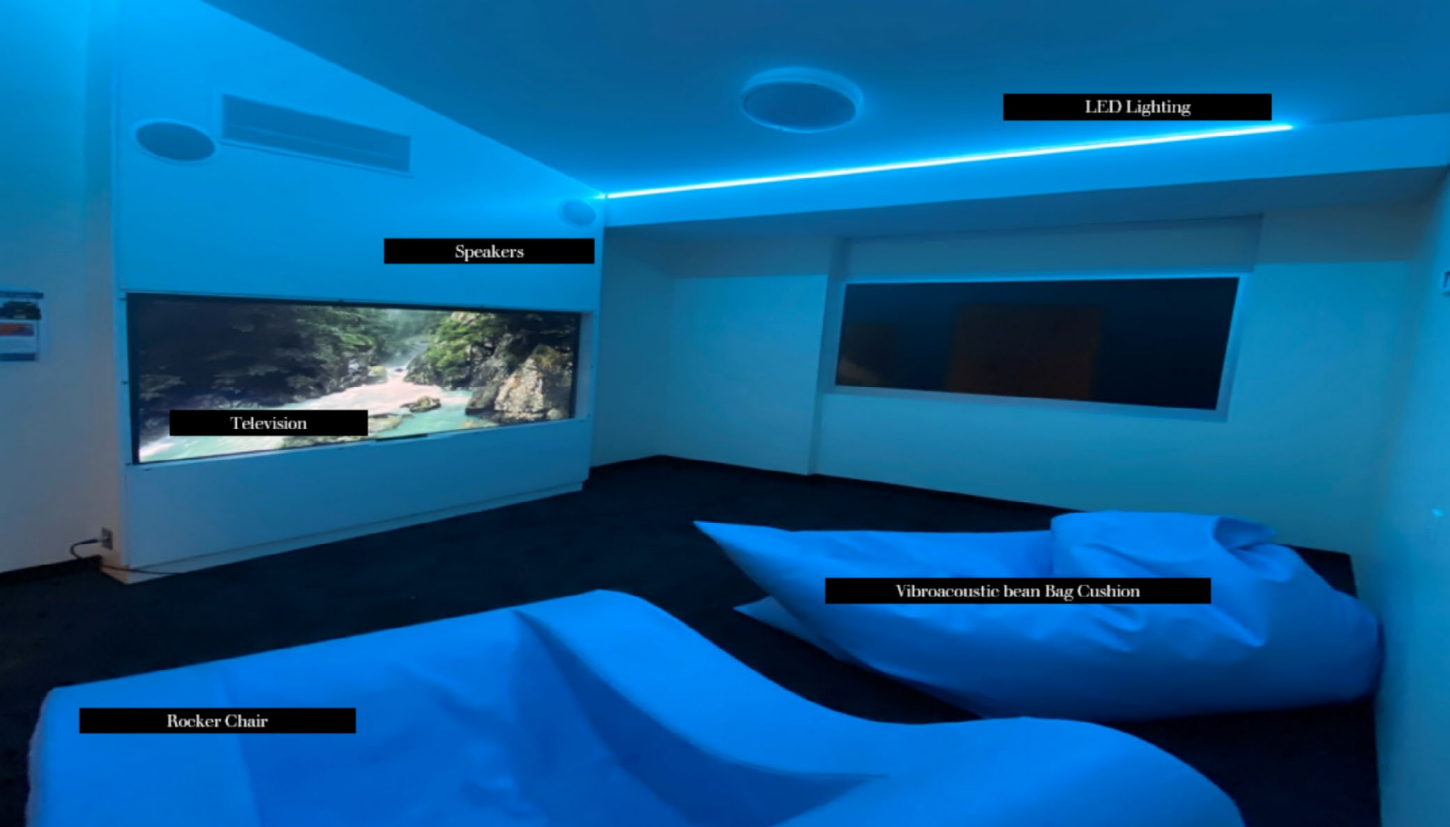
Sensory room image.

The sensory room aimed to support consumers to self‐regulate, improve care experience and support engagement in therapeutic activities. The room was fitted with specialised integrated sensory technology including an integrated sound, visual and lighting system, and a vibroacoustic beanbag and rocking chair that could be customised to suit sensory preferences. The room was intended to be available 24 h and be self‐initiated (consumer led) or staff initiated, and used for up to 30 min. Figure 1 provides an image of the sensory room.

**FIGURE 2:**
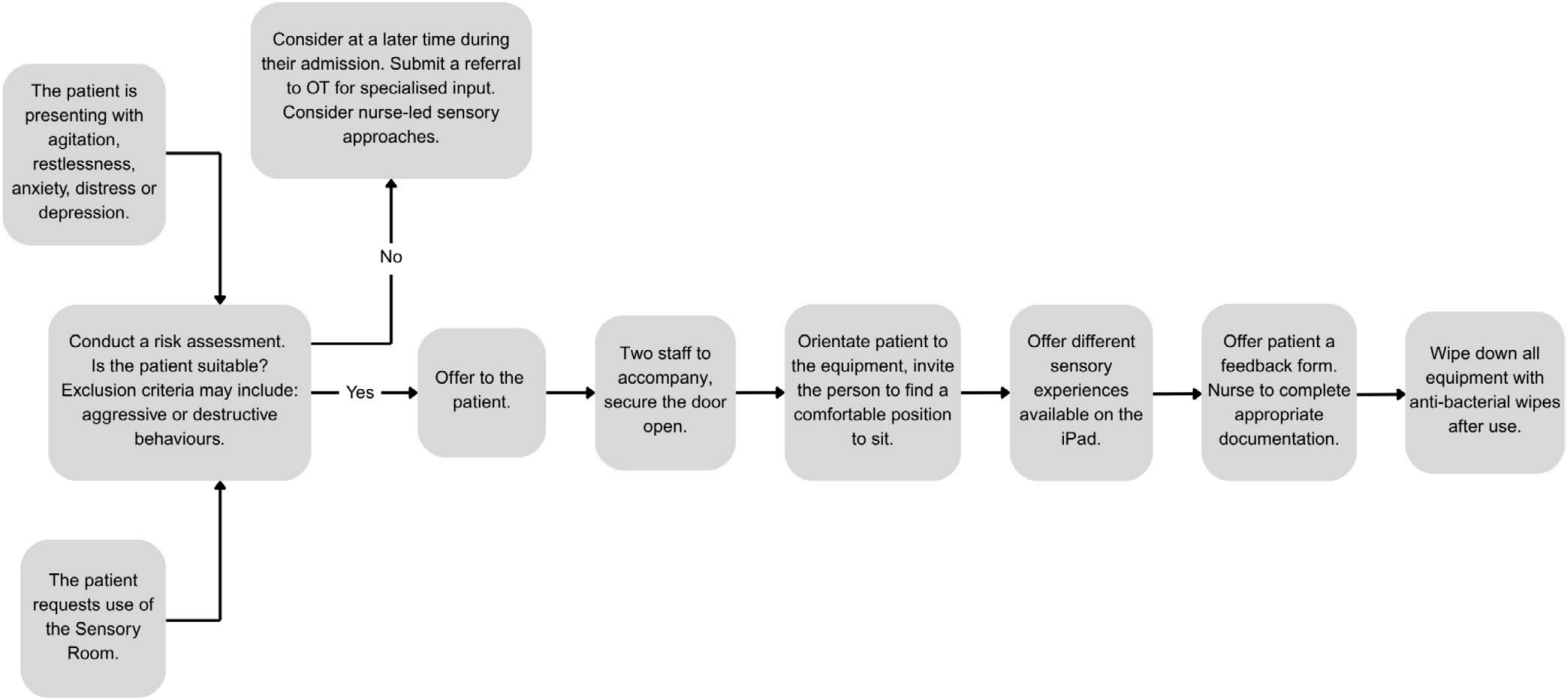
Sensory room use protocol.

The protocol for use, developed by nursing, occupational therapy and medical staff, stated that due to its location, two clinicians must remain in the sensory room with individuals throughout the session with the door remaining open (see Figure 2).

### 2.5 Implementation

Implementation was driven by a multidisciplinary steering committee (medical, nursing and allied health) led by an occupational therapist over 6 months. Educational materials for staff and consumers were developed, including written content and a video explaining the sensory room's purpose and use. Multiple in‐person training sessions were run with nurses about sensory approaches and the sensory room equipment and occupational therapists co‐facilitated sessions. Nursing staff were encouraged to complete a sensory approaches e‐learning programme.

### 2.6 Participants and Recruitment

Consumers and staff (from any discipline) who had trialled the sensory room were recruited from the PICU for interviews about their experiences. Participant information sheets were provided with details regarding consent and the right to withdraw from the study at any stage without consequences. Staff were invited or self‐nominated to participate. Consumers were approached by a staff member who was familiar to them and provided with information about the project to ascertain their interest in participating. Participation was voluntary, with no reason for excluding any consumers or staff members. Signed consent was obtained from all participants. A waiver of consent was approved by the HREC committee to access de‐identified data pertaining to incidents of seclusion and restraint.

### 2.7 Data Collection

Data pertaining to PRN and seclusion rates were collected at three time points. Firstly, prior to implementation, and then 10 and 17 months following implementation of the sensory room. At each time period, monthly data for two consecutive months were collated (prior to implementation: September and October 2021; 10 months following implementation: September and October 2022; and 17 months following implementation: May and June 2023). For PRN use, a single event of giving PRN was counted as one, even if multiple medications were given. Data regarding PRN use and seclusion episodes were collected by nursing staff working on the unit from medical records and de‐identified. Occupied bed day data for the unit were used to convert PRN use and seclusion episodes into ‘episodes per 1000 occupied bed days’ rates to allow for accurate comparison over different time periods.

Qualitative data were collected through individual interviews with consumers and staff members who had utilised the sensory room using a semi‐structured interview approach. Interviews were conducted by an investigator known to the consumers (PW) and staff (AH) in a private section on the ward with consumers and in an interview room for staff. Interviews with clinicians lasted for 30 min on average and 15 min with consumers were audio recorded and transcribed verbatim for analysis. See Appendix S1 for the interview questions. Two members from the mental health services consumer advisory group reviewed and edited the interview questions.

### 2.8 Analysis

#### 2.8.1 Quantitative Data

PRN and seclusion rates were calculated for each time period (prior to implementation; 10 months following implementation and 17 months following implementation) and reported as means and standard deviations. These were then examined for differences over time using an analysis of variance (ANOVA) procedure. In the ANOVA, monthly rates for PRN and seclusion episodes were assigned to the relevant time period and then analysed for differences between the three time periods.

#### 2.8.2 Qualitative Data

The research team familiarised themselves with the data and identified initial codes, with all transcripts coded by two researchers. The researchers continued to meet throughout the analysis process to review and refine the codes and discuss the development of themes which were generated through deductive analysis against the TDF (Cane et al. 2012) and COM‐B (Michie et al. 2011). Data were managed using NVivo (QSR International, released March 2021). As two members of the research team also participated in an interview, data from these interviews were coded by other team members. During the analysis and write‐up, the researchers' discussed how personal biases, assumptions and experiences could be influencing the interpretation of the data.

### 2.9 Positionality

The research team included four occupational therapists (two of whom are clinician researchers experienced in knowledge translation research), a mental health nurse and a nurse researcher. Four research members were involved in supporting the implementation and uptake of the sensory room in this study setting. All researchers are committed to supporting the uptake of best practice in clinical settings.

## 3 Results

Interviews were conducted with 12 staff members (10 nurses, 2 occupational therapists) and 8 consumers (6 male and 2 female).

### 3.1 Quantitative Results

Results for PRN and seclusion rates are presented in Table 1. The rate of PRN use remained relatively stable over the three time points (*F*(2, 3) = 0.23, *p* = 0.807). While there was a decrease in seclusion use from 75.0 per 1000 occupied bed days prior to implementation to 12.8 at the first follow‐up, this change did not reach statistical significance (*F*(2, 3) = 0.45, *p* = 0.677).

**TABLE 1:** PRN and seclusion rates over time.

Period	Month	OBD	PRN episodes	PRN rate per 1000 OBD^a^	PRN rate per period	Seclusion episodes	Seclusion rate per 1000 OBD^b^	Seclusion rate per period
Prior to implementation	September 2021	224	201	897.3		2	8.9	
Prior to implementation	October 2021	229.3	229	998.5	948.5	32	139.5	75.0
10 months following implementation	September 2022	232.3	271	1166.4		5	21.5	
10 months following implementation	October 2022	237	206	869.2	1016.3	1	4.2	12.8
17 months following implementation	May 2023	223	221	991.		20	89.7	
17 months following implementation	June 2023	207	222	1072.4	1030.2	0	0.0	46.5

Abbreviations: OBD, occupied bed days; PRN, pro re nata medication.

^a^ ANOVA for between‐period comparisons did not show significant differences between PRN rates across the three different periods (*F*(2, 3) = 0.23, *p* = 0.807).

^b^ ANOVA for between‐period comparisons did not show significant differences between seclusion rates across the three different periods (*F*(2, 3) = 0.45, *p* = 0.677).

### 3.2 Qualitative Findings

The prominent barriers and facilitators of sensory room use in the PICU—from the perspectives of staff and consumers—are presented below. Fourteen sub‐themes were identified across seven TDF domains, including 10 facilitators and seven barriers, which were then mapped to the COM‐B. Most facilitators (5/10) were themes that related to staff and consumer motivation to use the sensory room, and most barriers (4/7) related to limited staff opportunity to facilitate use. See Table 2 for a summary.

**TABLE 2:** Barriers and facilitators of sensory room use.

Capability			
Knowledge	+	Staff knowledge of sensory modulation	B/F
Skills	+	Staff technical skills to use software	B/F
Behavioural regulation	+++	Self‐regulation enhanced engagement	F

Opportunity			
Environmental context and resources	+	Changing workforce	B
	+++	Dependency on staff for access	B
	+	Location of the room	B
	+	Limited variety of equipment	B
	++	A safe space	F
Social influence	++	Supportive staff influence use	F

Motivation			
Professional role and identity	+	Variation in staff responses regarding role	B/F
Optimism	++	Consumer motivation to trial the sensory room	F
Beliefs about consequences	+	Positive staff perceptions and experiences	F
	++	Consumers' valued the space	F
Intentions	+++	Consumer and staff intention to use	F

*Note:* +, discussed by only staff; ++, discussed by only consumer; +++, discussed by staff and consumer.

Abbreviations: B, barrier; F, facilitator.

### 3.3 Capability

Two of the three sub‐themes (relating to staff knowledge and skills) were both facilitators and barriers to uptake of the sensory room; however, the impact on consumers ability to self‐regulate was a clear facilitator to uptake.

#### 3.3.1 Knowledge

##### 3.3.1.1 Staff Knowledge of Sensory Modulation

Limited nursing staff knowledge and understanding of sensory modulation and sensory rooms value was a prominent barrier to implementation. Limited knowledge meant that nursing staff found it challenging to explain sensory modulation to consumers, thus limiting consumers' understandings of the potential benefits.

…A lot of people seem to think that sensory modulation is just fidget toys, and they fail to understand how every moment of our day…we are receiving sensory input and how important sensory input [is] to how we feel, how we think and how we behave. So, it's definitely lack of staff knowledge (Staff IV 6)

#### 3.3.2 Skills

##### 3.3.2.1 Staff Technical Skills to Use Software

The perceived complexity in operating the installed software was identified as a barrier to use. Nurses reported limited technical skills required to operate the software due to limited familiarity and experience. However, staff reported that after using the technology they were confident in supporting consumers to use the room.

Staff consumer knowledge – yes, if you haven't used it before, people are very reluctant. Especially the computer software which drives it (Staff IV 1)

My experience definitely plays a role, I am comfortable now taking people in the room than I was when I first started…I think that's a normal part of the process of adjusting to a new workplace. (Staff IV 6)

Staff knowledge and technical skills were also key facilitators for use of the sensory room. Most staff emphasised that staff education and training to equip them with the required knowledge and skills would facilitate use. They emphasised the need for ongoing support, education and training for new and/or casual nursing staff and written materials for staff and consumers. Ensuring consistency in what staff communicated to consumers about sensory rooms was considered to increase consumer engagement.

There was no training for new people. I just learnt it by trial and error…If there is something…to read…what this sensory room is so we can sit with that consumer, explain to them that what is expected…and how we use it, and what you can do to help getting more engaged, I think that will help (Staff IV 1)

There are ways to support new staff to implement that intervention to help them understand not only the intervention but why everybody has to right to use that intervention that is actually going to help their mental state. (Staff IV 6)

#### 3.3.3 Behavioural Regulation

##### 3.3.3.1 Self‐Regulation Enhanced Engagement

Consumers reported that experiencing the sensory room was a ‘whole of body experience’ that positively impacted on their mood and sleep and helped them manage their emotions if they felt ‘sad or upset or frustrated’. The use of music was specifically highlighted as helpful in self‐regulating and calming. These positive experiences facilitated consumers uptake and utilisation of the room.

It brought out a lot of emotions, relaxed and calmed me.…I had about 8 hours sleep for the first time ever in the last 2 to 4 weeks. (Consumer IV 5)

They make you sort of feel different…you feel something all over the body, it makes the hairs on the back of your neck stand up, and you know it makes you feel like you're doing something. (Consumer IV 1)

A few staff reported that consumers' experiencing heightened levels of distress when using the sensory room was a barrier to use and potential effectiveness.

The only example I have heard is that someone's gone up and …they became more overwhelmed with whatever they were looking at on the screen. (StaffI V 8)

Other staff considered use of the sensory room as having the potential to support development of self‐regulation skills.

A lot of people don't have the skills to self‐regulate, but being able to provide them with the opportunity to develop those skills is really important. (StaffIV 12)

Consumers expressed the value of being asked by staff to use the room, acknowledging that they may be feeling overwhelmed, however not seeing this as a barrier to them using the room.

Sometimes things can be overwhelming, and I just shut down or just, like be silent…or walk away… I don't always think of things like that and so that was good because they ask you…what things you like. (Consumer IV 4)

I think it's good for people like me, like sometimes my emotions is hard to keep… in the standard level. If I go to the sensory room, can put my emotion back. (Consumer IV 3)

### 3.4 Opportunity

Four prominent barriers related to the Environmental Context and Resources. Facilitators included consumers identifying the sensory room as a safe space and having staff support access.

#### 3.4.1 Environmental Context and Resources

##### 3.4.1.1 Changing Workforce

Staff reported the challenges in implementing a new intervention that came from a changing workforce. It was difficult for new and/or casual nursing staff to acquire the required knowledge and skills to use the room.

A lot of new casual staff…probably wouldn't have any knowledge about the sensory room…if we could organise some more education sessions on that would be really beneficial. (Staff IV 7)

##### 3.4.1.2 Dependency on Staff for Access

Two staff members were required to facilitate consumer access to the sensory room, which was a prominent barrier to its use. Both staff and consumers expressed frustration with this requirement.

The staff resources will not be enough there because we need 2 nurses and then it's a PICU area, we can't leave all the nurses alone in this nursing station. So, it's hard for us to get them to the sensory room sometimes, it's one of the very strong barriers. (Staff IV 4)

It shouldn't be like you have to wait like 2 days and 3 hours and 20 minutes and 10 minutes and 10 seconds to get in there and then you have to wait for 2 nurses and 2 nurses and 2 nurses and all that. (Consumer IV 6)

##### 3.4.1.3 Room Location

All staff reported that the location and the sensory room protocol (e.g., the requirement for two staff and need to leave the door open) were major barriers to utilisation. Noise from the surrounding environment was reported to disrupt the sensory experience of users and reduce the effectiveness of the intervention, and staff highlighted the need for better accessibility of the room from the ward.

What is ineffective at the moment is obviously the location of the sensory room is quite challenging. Having to keep the door open is difficult because you are not getting that immersive sensory experience because you can hear all this noise coming from the ward…it's really disruptive. (Staff IV 12)

Staff reflected that the poor location then further impacted on staff motivation to utilise the sensory room.

The biggest barrier would be the location but then that makes the other barrier which is the staff willingness to participate and to learn something new and to use a new intervention it just highlights it even more. (Staff IV 6)

##### 3.4.1.4 Limited Equipment Variety

Some staff reported that there was a limited variety of sensory equipment, which they believed impacted on consumers' motivation to use the sensory room and thereby reduced the effectiveness of the intervention.

If there are more equipment in the room they will be more…motivated (Staff IV 4)

##### 3.4.1.5 A Safe Space

Most consumers reported that the sensory room provided them with an opportunity to withdraw from the stressful ward environment.

If I was sad or upset or frustrated with another patient, it would be a good place to sort of pull back and disassociate from the rest of the environment. (Consumer IV 1)

#### 3.4.2 Social Influence

##### 3.4.2.1 Supportive Staff Influenced Use

Consumers reported that they were positively influenced by staff recommendations and encouragement to use the room. Several consumers spoke about the benefits of repeated exposure to the room over time.

I didn't – initially the first time I didn't choose, it was just stuck upon me and – but now that the third time is hopefully coming to fruition soon. (Consumer IV 6)

The first time the staff invited me, the second time I asked (Consumer IV 1)

### 3.5 Motivation

Five facilitators were identified across four TDF domains related to Motivation, with staff views regarding sensory approaches the only barrier.

#### 3.5.1 Professional Role and Identity

There was variation in staff responses regarding their views about whether engaging in sensory approaches sat within their role. Staff were required to facilitate consumer sensory room use. As such, positive staff attitudes of the intervention were pivotal in its uptake. One staff member commented that the sensory room use could assist staff in their work.

Staff will play a very significant role. Their attitudes and their involvement and choosing the sensory modulation as a tool to, to help them do their day‐to‐day work will correctly push things in the right direction. (Staff IV 9)

Some nursing staff viewed facilitating sensory room use as the role of occupational therapist.

I think there's probably some nursing staff that have never actually been into that room, despite it being part of their job to potentially utilise it. (Staff IV 10)

There is quite a divide in terms of somebody who has more knowledge about the room and has used the room will have their little spiel that is really helpful, and then there is new staff that have just come in and they are just like oh it's a room with a TV and the OT is going to take you. (StaffI V 12)

#### 3.5.2 Optimism

##### 3.5.2.1 Consumer Motivation to Trial the Sensory Room

Most consumers had never experienced using a sensory room prior to this unit; however, they were open to trialling. Following room use, consumers' positive experiences and understanding of the potential benefits facilitated further utilisation. Several consumer participants reported that their experience of the room and its benefits exceeded their expectations. Consumers emphasised the value of improving awareness about the sensory room to facilitate its utilisation among patients in the PICU.

First…I didn't know what I was going in to. And then I realised what I was going into, and seeing what I saw it was just…real good. (Consumer IV 2)

Just introduce it more…make it a bit more aware to the other patients. (consumer IV 8)

It's a surprise because I…get into a room like this and…it's different, totally different (Consumer IV 3)

#### 3.5.3 Beliefs About Consequences

##### 3.5.3.1 Positive Staff Perceptions and Experiences

Most staff reported that using the sensory room would result in positive benefits for consumers. Staff emphasised that room use resulted in reduced agitation and enhanced calm, relaxation, sensory regulation and overall enhanced well‐being. Staff attitudes impacted on sensory room use as staff who did not believe in the benefits of the intervention did not facilitate its use. Staff who were favourable considered the room an opportunity for empowering consumers to develop skills to self‐regulate their emotions.

A lot of people don't have the skills to self‐regulate, but being able to provide them with the opportunity to develop those skills is really important I think. (Staff IV 12)

Staff acknowledged the challenges in identifying the ‘best time’ to offer the sensory room to consumers:

If you can engage with someone before they've reached that level of, you know, there's just no further meaningful engagement, which often can mean having to get a code black…medication, seclusion. If you can get them before that and…going in and listening to music, it can really, help someone who is starting to escalate. (Staff IV 7)

Staff highlighted that sensory room effectiveness depends on consumer engagement and their beliefs about its benefits.

It depends on person to person… I've seen a person who is agitated like 10 out of 10 coming [out] 3, and sometimes even without using any…medication. So, it depends if they engage. (Staff IV 1)

They might even come and say I'm starting to be agitated, can we go and again, sometimes unfortunately we can't in that moment when they are escalating…But yes, I guess they will try to go back when they appreciate the benefit. (Staff IV 11)

##### 3.5.3.2 Consumers' Valued the Space

Consumers reported numerous positive effects of the intervention and highlighted the value of the sensory room for them within the PICU setting. Prominent benefits reported by consumers were relaxation, reduced anxiety, anger control, enhanced emotional awareness, multi‐sensory stimulation, serenity and emotional regulation. They also highlighted the sensory room's uniqueness.

Just got me into a peaceful state of mind, less anxiety and just took my bad thoughts away to just calming me down I suppose. (Consumer IV 8)

#### 3.5.4 Intentions

##### 3.5.4.1 Staff and Consumers' Intention to Use

Those staff who considered the sensory room to be an effective intervention regretted the underutilisation and were motivated to use the room to benefit consumers. Positive consumer experiences and their desire for repeated use further encouraged staff to facilitate sensory room use as part of care. Staff also reported that consumers would request repeated use after experiencing positive outcomes.

I've always found that it differs the amount of time that somebody might want to spend in there, but I've found that the patients that I've taken in…sometimes it's hard to get them out really, but I haven't found that anyone has had to leave because it's causing more distress or they're not enjoying the experience. (Staff IV 12)

Consumers expressed their desire to use the sensory room more often, if possible, daily.

I'm getting discharged; otherwise, I would try to use it every day. (Consumer IV 1)

## 4 Discussion

This study reports on an interdisciplinary quality improvement initiative to implement a sensory room in a PICU. The intent was to offer a safe space to support consumers self‐regulate distressing emotions, and for staff, an alternative option to more restrictive practices. There were no changes in restrictive practice use (as measured by PRN use, medication and seclusion) in our study. Various factors could influence this lack of change. Existing practices and policies regarding PRN medication and seclusion might be deeply ingrained in the service culture, making it challenging to observe immediate changes with the introduction of novel interventions. Nurses continue to report PRN medication as the most effective strategy to support individuals experiencing an exacerbation in their psychotic symptoms (Wong and Müller 2023), which includes most consumers admitted to a PICU. A recent systematic review concluded that there is limited evidence as to whether sensory rooms are effective at reducing seclusion, restraint or violence; however, they are likely to support a reduction in patient distress (Haig and Hallett 2023). This last finding is still important and aligned with our qualitative findings.

In clinical practice, there are challenges in implementing novel interventions; thus, we were interested in understanding the barriers and facilitators to utilising the sensory room in a PICU. These factors were mapped against the domains of capability, opportunity and motivation for changing behaviour, which are considered essential components for changing care practices (in this instance, promoting and using a novel intervention). In this study, change in behaviour referred to staff offering and facilitating sensory room use, and consumers openness to, and uptake of the room. Below, we discuss some key findings alongside strategies that could enhance sensory room uptake in an acute mental health care setting.

### 4.1 Capability

For a change in behaviour there must be the ‘capability’ to do it. Our findings demonstrated that consumers who were experiencing high levels of distress and agitation had the capability to meaningfully engage and benefit from the sensory room. However, consumers in this study were reliant on staff to access the room. Both staff and consumers reported limited knowledge of the purpose and potential benefits of the room. Staff also reported limited skills in using the equipment in the room. Limited staff capability impacted on their ability and confidence to facilitate consumers use of the room. Staff indicated the need for regular educational sessions and structured training to build their knowledge and confidence, and easily understandable pamphlets to enhance consumer awareness and engagement. Consumer and staff understanding of sensory modulation has been found to be essential for uptake (Andersen et al. 2017; Barbic et al. 2019) with training a key factor associated with sensory room use (Wright et al. 2022). Education should be tailored to the audience, for example Barbic et al. (2019) found that service users wanted more knowledge about the specific sensory modalities in the room, while nursing staff wanted more education on the evidence for its benefits. Our study highlighted that enhanced staff knowledge and skills facilitated use. Increased familiarity with the sensory room and observations of its benefits increased staff confidence and motivation to use the room. This aligns with findings from another study where staff initially described negative and positive expectations of sensory rooms, and then after repeated use reported more positive experiences, including increases in consumers' self‐confidence, emotional self‐care and well‐being (Björkdahl et al. 2016).

### 4.2 Opportunity

For a change in behaviour, there must be the opportunity for a behaviour to occur in terms of a conducive physical and social environment. This includes factors such as physical accessibility, social acceptance and having sufficient time. The major barriers to uptake of the sensory room related to accessibility and staffing, whilst social influences (staff encouraging consumers) were a facilitator.

Recognising environmental stressors and exploring strategies that can help individuals adapt to these demands is a core component of sensory modulation (Champagne and Sayer 2014). Whilst this was the project intent, the room location and the requirement for two staff to be present to facilitate use were major barriers to its uptake. Removing two nursing staff from five total nursing staff was a barrier that could not be safely overcome. Consumers have highlighted the importance of being able to choose when and how they use a sensory room (including length of time and alone or with staff support) (Hedlund Lindberg et al. 2019). Nursing staff have reported feeling fear and blame regarding the challenges they face in reducing restrictive practice use in acute mental health care settings (Muir‐Cochrane et al. 2018), so alternative strategies need to be feasible. The location resulted in the underutilisation of the sensory room and negatively impacted on staff perceptions of its value. Explorations of staff concerns regarding risk and specifically, the potential for risk aversion, are important. A recent scoping review exploring the use of sensory rooms in adult mental health care settings reported both independent and staff‐supported use (Doroud et al. 2025). Lower supervision levels have been found to result in increased usage of sensory rooms (Forsyth and Trevarrow 2018) with staff reporting fewer concerns about consumers independently using sensory rooms over time (Björkdahl et al. 2016; Hedlund Lindberg et al. 2019).

The environment has been acknowledged to be a barrier to the range of initiatives that seek to reduce and/or eliminate restrictive practice use (Carlson and Hall 2014). Studies reporting on environmental barriers that impact on sensory room utilisation have emphasised a need for the environment to feel less ‘clinical’, be located in a quiet place on the ward allowing for uninterrupted sessions, be easily accessible, well equipped and maintained (Wright et al. 2020; Haig and Hallett 2023; Wiglesworth and Farnworth 2016). Altering the environment and providing soothing approaches can assist consumers' ability to self‐regulate emotions (Kandlur et al. 2023); however, this can be challenging when retrofitting an established build, as demonstrated in this study.

### 4.3 Motivation

For a change in behaviour there must be sufficient ‘motivation’ to do it. In this study, the main facilitators to sensory room use related to staff and consumers' positive experiences. Consumers and staff reported that the room had positive impacts on individuals' mood, sleep and emotional regulation. Consumers also spoke about using the room as a positive space where they could escape from the busy and stressful ward environment. These findings align with other research into consumer and staff experiences of sensory rooms, which include supporting emotional regulation, promoting self‐management, reducing distress and positively impacting on patient experiences (Haig and Hallett 2023).

There were mixed responses from staff regarding their professional role and identity, with some nursing staff identifying occupational therapists as the key facilitator for the sensory room. Whilst sensory modulation is a core competency of occupational therapists, nursing staff regularly support the use of sensory rooms in acute mental health care settings (Haig and Hallett 2023; Doroud et al. 2025). Other studies examining the uptake of sensory approaches in mental health settings found they were used with a limited proportion of consumers (Wright et al. 2022), and highlight the need for interdisciplinary uptake (Dawson et al. 2022; Scanlan and Novak 2015) so this is an important barrier to address. Staff have highlighted the importance of training (Doroud et al. 2025) alongside opportunities to experience sensory approaches as enhancing motivation (Forsyth and Trevarrow 2018).

In Table 3, we present specific interventions to consider when implementing novel psychosocial interventions in a PICU. These interventions were developed by the research team from the findings and have been mapped against the COM‐B (Michie et al. 2011).

**TABLE 3:** Intervention recommendations for sensory room use based on COM‐B.

	Capability			Opportunity		Motivation			
	Knowledge	Skills	Behavioural regulation	Environmental context and resources	Social influence	Professional role and development	Optimism	Beliefs about consequences	Intentions
**Education**									
1. OT to facilitate regular staff educational sessions on sensory approaches, use of the room.	✔					✔		✔	✔
2. Co‐designed pamphlet for consumers on admission regarding sensory approaches and room access.	✔								
**Training**									
1. OT to co‐facilitate sessions with nursing staff.		✔		✔		✔			
2. Incorporate training into staff orientation.		✔		✔		✔			
**Persuasion**									
1. Encourage staff to share experiences of supporting consumers to use the sensory room.					✔		✔	✔	✔
2. A feedback board for consumers to comment on experiences of sensory approaches and the sensory room.					✔		✔	✔	✔
**Environmental restructuring**									
1. Ensure the sensory room is in an accessible location with minimal noise disruptions.				✔					
2. Ensure sensory room use is incorporated into established ward routines (e.g., handover, clinical reviews).				✔					
**Modelling**									
1. Establish interdisciplinary leads to model, support and encourage staff to use.					✔		✔	✔	✔
2. Explore opportunities for consumers to share experiences of sensory room use (e.g., ward community meetings).					✔		✔	✔	✔
**Enablement**									
1. Co‐design protocols for sensory room use to support independent use			✔	✔			✔		
2. Encourage staff to use the sensory room themselves to get a personal experience.			✔	✔			✔		

### 4.4 Limitations

The study was conducted in a single PICU within a specific psychiatric facility, which may limit the transferability of findings to other settings with different infrastructure or clinical practices. Further limitations include lack of consumer involvement in the design and conduct of the project, inability to record frequency of use of the sensory room and lack of a comparison group. Additionally, as this study was based on a small PICU, there is a risk that variations in PRN use and seclusion rates could have been substantially influenced by one or two individuals on the unit. This has the potential to have obscured otherwise positive changes that may have occurred following the implementation of the sensory room.

## 5 Conclusion

Findings highlight the barriers and facilitators to sensory room use in a PICU and strategies and considerations for more effective use. Sensory room use was valued by both consumers and staff in a PICU, with staff and consumer motivation key facilitators. Prominent barriers related to staff opportunity to facilitate use of the sensory room and are acknowledged as significant limitations that impacted on staff ability to support consumers to use the room. Suggested strategies for successful implementation of a sensory room and other novel psychosocial interventions are informed by the COM‐B.

## 6 Relevance for Clinical Practice

This real‐world study provides various learnings for use in clinical practice. The application of implementation frameworks in this study provides theory‐based barriers and facilitators and suggested future implementation strategies for sensory rooms and other novel interventions in acute mental health care settings. Nurses are the primary workforce in acute mental healthcare settings and are key facilitators to consumer sensory room uptake. Identification of nurse champions to promote uptake of sensory approaches and ongoing training and education for consumers and staff would increase successful uptake.

## Author Contributions

S.D., A.H. and N.J. contributed to the conception and design of the study. A.H. and P.W. led the data collection. All authors participated in the analysis and interpretation of results. S.D., L.B. and J.N.S. wrote the first draft of the manuscript with all authors reviewing and editing the final manuscript.

## Ethics Statement

Ethics approval for the study was received from Southern Adelaide Clinical Human Research Ethics Committee (2022/HRE00122).

## Conflicts of Interest

The authors declare no conflicts of interest.

## Supporting information




Appendix S1.

## Data Availability

The data that support the findings of this study are available from the corresponding author upon reasonable request.
