# Supplementary material for: Implementation of a Sensory Room in a Psychiatric Intensive Care Unit: A Mixed‐Methods Study
Source: Int J Ment Health Nurs. 2025 Jul 31;34(4):e70103. doi: 10.1111/inm.70103 (PMC12314344; doi:10.1111/inm.70103)
Supplement: Supplementary file 1 — Appendix S1. [file INM-34-0-s001.docx]

**Research questions for consumers**

Purpose of interview-

We are interested in understanding your experiences of the sensory room in PICU. The interview will take about 15 minutes. If you agree, I will record our discussion so that it can be typed up as a record for the research team to read through and understand your and other people’s experiences of the room. If you prefer not to have the interview recorded, I will make notes to record the conversation. You can also stop the interview at anytime you wish.

Before we start, can you tell me a little about yourself? (How long you have been on the ward, what are your hopes for the future)

Can you tell me about how you were introduced to the sensory room? (By whom? When?)

I’d like to ask you a few questions about your experience of using the sensory room:

- Have you used it and how many times?
- When did you use it? (morning/afternoon/evening)
- Did you or staff initiate using the sensory room?

Can you tell me about why you chose to use the sensory room?

Can you tell me what you like (find helpful) about using it?

Can you tell me what you don’t like (find unhelpful) about using it?

Was there anything that stopped you using the room more often?

Is there anything that would make you feel more likely to use the room more often? (If so, what?)

Did using it work as you expected? Was there anything you didn’t expect?

Have you ever used sensory approaches or a sensory (or comfort) room before this admission? Can you tell me a little about your experiences of this?

Do you have any ideas on what might be useful to introduce the sensory room to others?

To what extent do you feel using the sensory room has supported your distress and agitation? (Leave if already addressed).

Is there anything else you would like to add about your experience of using the sensory room?

Thank you for participating in this research project.

**Research questions for staff**

We are interested in understanding your experiences of the use of the sensory room with consumers on the unit. The interview will take about 30 minutes. If you agree, I will record our discussion so that it can be typed up as a record for the research team to read through and understand your and other staff experiences of the room. If you prefer not to have the interview recorded, I will make notes to record the conversation. You can also stop the interview at anytime you wish.

Can you tell me about your prior experience of using sensory approaches (including comfort rooms/sensory rooms) with consumers?

Can you tell me about any occasion/s that you’ve offered (or observed a consumer using) in the sensory room?

- What happened?
- What role did you play?
- What (if any) outcomes did you observe? / changes in consumers presentation?
- What would prompt you to offer (or suggest) someone trial the room?

Has there been anything you expected or didn’t expect regarding use of the sensory room by consumers?

When the sensory room hasn’t worked for consumers, in what respect did it not work and why?

We are interested in potential barriers and facilitators to using the sensory room with consumers.

What things about the person (yourself and the consumer) or environment do you think made using the sensory room effective or ineffective?

Do you feel that ______ plays a role and how?

- staffing
- workplace procedures
- staff or consumer knowledge
- consumer characteristics
- location
- prior experience (yours or theirs)

Do you have any ideas on what might be useful when introducing the sensory room to consumers?

To what extent do you feel use of the sensory room supports consumers self-management of distress and agitation?

Is there anything else you would like to add about your experience of using, or not using the sensory room with consumers?

Thank you for participating in this research project.
